# Evaluation of Growth, Viability, Lactic Acid Production and Anti-Infective Effects of *Lacticaseibacillus rhamnosus* ATCC 9595 in Bacuri Juice (*Platonia insignis*)

**DOI:** 10.3390/foods10030603

**Published:** 2021-03-12

**Authors:** Yasmim Costa Mendes, Gabrielle Pereira Mesquita, Gabrielle Damasceno Evangelista Costa, Ana Carolina Barbosa da Silva, Ester Gouveia, Maria Raimunda Chagas Silva, Valério Monteiro-Neto, Rita de Cássia Mendonça de Miranda, Luís Cláudio Nascimento da Silva, Adrielle Zagmignan

**Affiliations:** 1 Laboratório de Patogenicidade Microbiana, Universidade Ceuma, São Luís 65075-120, Brazil; yasmimc.mendes@yahoo.com.br (Y.C.M.); gabi_mesquita8@hotmail.com (G.P.M.); gabrielledamasceno.nutri@gmail.com (G.D.E.C.); carolinabsilva07@gmail.com (A.C.B.d.S.); luisclaudionsilva@yahoo.com.br (L.C.N.d.S.); 2 Department of Antibiotics, Federal University of Pernambuco, Cidade Universitária, Recife 50670-901, Brazil; estergouveia@gmail.com; 3 Laboratório de Química Ambiental, Universidade Ceuma, São Luís 65075-120, Brazil; marirah@gmail.com; 4 Programa de Pós-Graduação em Ciências da Saúde, Universidade Federal do Maranhão, São Luís 65085-582, Brazil; valerio.monteiro@ufma.br; 5 Laboratório de Microbiologia Ambiental, Universidade Ceuma, São Luís 65075-120, Brazil; rita.miranda@ceuma.br

**Keywords:** lactobacilli, probiotic-based products, lactic fermentation, anti-infective agents

## Abstract

Fruit juices have been emerging as excellent vehicles for development of probiotic products due to their nutritional properties and presence of bioactive compounds. This work evaluated the growth and viability of *Limosilactobacillus fermentum* ATCC 23271 and *Lacticaseibacillus rhamnosus* ATCC 9595 in bacuri juice (*Platonia insignis* Mart., Clusiaceae). Both strains were able to grow in bacuri juice, without any supplementation. Viability was kept after 28 days of storage; however, growth was significantly higher for *L. rhamnosus* ATCC 9595 (7.40 ± 0.04 Log CFU/mL). Following this, the effects of bacterial inoculum and pulp concentration on growth and lactic acid production by *L. rhamnosus* ATCC 9595 were investigated using a central composite rotational design. The inoculum concentration was the main factor for obtaining the most favorable relation between growth and organic acid production (G/pH ratio). Among the tested conditions, those used in assay 6 allowed the best G/pH ratio (2.13) and higher lactic acid production (4.14 g/L). In these conditions, *L. rhamnosus* ATCC 9595 grown in bacuri juice showed the same resistance towards acidification or addition of lysozyme than when cultivated in MRS. Finally, the anti-infective effects of fermented and non-fermented juices were analyzed using *Tenebrio molitor* larvae infected by enteroaggregative *Escherichia coli* 042. The pre-treatment with supernatants of both fermented and non-fermented juices significantly increased the survival of *E. coli-*infected larvae. However, only the *L. rhamnosus*-fermented juice had protective effects when inoculated 2 h after infection. Collectively, the results obtained in this research allowed the basis for the development of a non-dairy probiotic product from bacuri juice.

## 1. Introduction

Functional foods containing probiotics have been demonstrating effectiveness to prevent or treat health problems, including digestive disorders, such as irritable bowel syndrome and necrotizing enterocolitis [1,2,3]. In particular, those formulations containing lactobacilli are recognized for their ability to modulate the human microbiota and induce anti-inflammatory and anti-oxidant effects [4,5,6].

Probiotics are commonly found in dairy foods, such as milks, yogurts and cheeses [4,7,8]; however, fruit juices may also be excellent vehicles for development of probiotic products due to their nutritional properties and presence of bioactive compounds (vitamins, phenolic acids, flavonoids and other anti-oxidant compounds) [9,10,11]. The use of fruit juices containing probiotics also gives opportunities to individuals with specific conditions (lactose intolerance, allergy to milk components and vegetarians) to benefit from the consumption of these bacteria [12,13].

Brazil is a country that has one of the largest repertoires of fruits in the world, constituting a comprehensive amount of tropical and exotic fruits with unique aromas and flavors [14]. An example is bacuri, the fruit of *Platonia insignis* Mart. (Clusiaceae), a medicinal plant popularly known in Brazil as bacurizeiro. Bacuri is a round fruit, with thick skin and a citrus-yellow color. It contains a very tasty viscous pulp, used in sweets, liqueurs and ice cream [15,16].

Some functional characteristics have been reported to bacuri pulp such as its anti-oxidant potential and α-glucosidase inhibitory action [17]. The fruit is also rich in bioactive compounds (for instance, citric acid, p-coumaric acid and terpenes) [17,18], in addition to the presence of sugars (glucose, fructose and sucrose), vitamins (C and E) and metals (Na, K, Ca, Mg, P, Fe, Zn and Cu) [19]. All together, these characteristics make bacuri juice an interesting alternative vehicle for the development of fermented products, where the chemical constituents present in bacuri juice could potentiate the beneficial effects of probiotic bacteria.

In this context, the present work evaluated the growth of *Limosilactobacillus fermentum* ATCC 23,271 and *Lacticaseibacillus rhamnosus* ATCC 9595 in bacuri juice. These species were previously classified as *Lactobacillus fermentum* and *Lactobacillus rhamnosus*, respectively. However, the genus *Lactobacillus* was recently revised based on genomic analysis resulting in the distribution of the species in 25 genera [20]. Following this, the influence of pre-inoculum density and pulp concentration on the growth of *L. rhamnosus* ATCC 9595 and lactic acid production in the juice were evaluated by central composite rotational design (CCRD). Finally, we analyzed the protective effects of the fermented juice in a model of infection induced by enteroaggregative *Escherichia coli* (EAEC) in *Tenebrio molitor* larvae.

## 2. Material and Methods

### 2.1. Origin and Maintenance of Probiotic Strains

The strains used in this study were obtained from the Microbial Collection of the Ceuma University. The strains were kept refrigerated at −80 °C. For the experiments with *L. fermentum* ATCC 23,271 and *L. rhamnosus* ATCC 9595, the aliquots were activated in MRS broth (De Man, Rogosa and Sharpe [21]).

### 2.2. Fruit Collection and Pulp Characterization

The plant material from *P. insignis* was collected in the Cerrado Maranhense region, in the south of the Maranhão state. Afterwards, the samples were identified in the Ático Seabra Herbarium from the Federal University of Maranhão by Prof. Dr. Eduardo B. de Almeida Jr (voucher specimen number 11.540). The fruit pulp was manually removed and stored at −20 °C until its preparation.

Analyses to determine the proximate composition of *P. insignis* were carried out in triplicate [22]. The composition of crude fat was determined by the Soxhlet method, and determination of moisture and ashes was performed as described in the physical–chemical methods for food analysis [23]. The amount of carbohydrates was indirectly determined by calculating the difference from the other constituents, using the equation: total carbohydrates = 100 − (moisture + ashes + proteins + lipids).

### 2.3. Initial Fermentation and Viability Tests under Refrigeration

In the initial fermentation tests, an aliquot of pulp (30 g) was dissolved in 250 mL of distilled water (concentration of 120 mg/mL). The pH of the juice was adjusted to 6.0 before sterilization [24,25]. In parallel, a pre-inoculum for each bacterium (*L. fermentum* ATCC 23271 or *L. rhamnosus* ATCC 9595) was prepared in MRS broth. Probiotics were grown at 37 °C under agitation (120 rpm). After 24 h, aliquots (1 mL) of each bacterial suspension, at an optical density of 600 nm (OD_600 nm_) of 1.0, were inoculated in bacuri juice or in an MRS broth. The flasks were incubated with shaking at 120 rpm for 48 h.

Quantification of bacterial growth was performed by plating on MRS agar. Serial dilutions were made in PBS solution after each determined period (0, 7, 14, 21 and 28 days of refrigeration). Then, the petri dishes were incubated for 48 h at 37 °C. The number of bacteria was determined by counting colony-forming units (CFU) and expressed in CFU/mL.

### 2.4. Effects of Inoculum Density and Pulp Concentration on Growth and Lactic Acid Production

The effects of inoculum density and pulp concentration on growth and lactic acid production were evaluated by central composite rotational design (CCRD). The two variables chosen were the inoculum concentration (x^1^) and the pulp concentration (x^2^), with a total of 10 assays (Table 1). After 48 h, the pH values and the microbial population were analyzed to determine the relationship between bacterial growth and pH (G/pH), and to ensure better production of lactic acid [26].

#### Quantification of Lactic Acid Content

The quantification of lactic acid was performed as described by Farias, Soares and Gouveia [26], using a Shimadzu high-performance liquid chromatograph system equipped with a quaternary pump coupled to a degassing system (DGU-20A5r). The apparatus contains an oven to control the column temperature (set at 28 °C) and an automatic injector (20 µL injection) with a diode array detector (SPD-M20A; range 190–800 nm). An ion exchange column (300 mm × 7.8 mm × 9 µm; Aminex^®^ HPX-87H, Bio-Rad, Hercules, CA, USA) was used. The elution was carried out isocratically with a mobile phase composed of 5 mM H_2_SO_4_ and with a flow of 0.6 mL/min. The software used was LC-Solutions manufactured by Shimadzu Corporation (Kyoto, Japan).

For each test, the concentration of lactic acid in an unfermented juice containing the same concentration of pulp (unfermented controls) was also detected. The production of lactic acid (g/L) was determined by the difference between the concentration of lactic acid in each fermented liquid and its respective unfermented control.

### 2.5. Effects of Juice on Bacterial Tolerance towards Lysozyme and pH

Tolerance tests were performed as described by Singhal (2010) with modifications. In the first trial, lysozyme (Sigma-Aldrich, St. Louis, MO, USA) was added to the MRS broth or to the juice (230 mg/mL of bacuri pulp) to reach a concentration of 300 µg/mL. An aliquot (100 µL) of each modified medium (MRS broth or bacuri juice containing lysozyme) was transferred to 96-well microplates. Subsequently, a sample of 10 µL of the fermented juice (fermented under the conditions of assay 6: 230 mg/mL of bacuri pulp and pre-inoculum at OD_600_ of 2.33) was added to each well. Similarly, in the acidity tolerance tests, the pH of the juice or medium was adjusted to reach pH values of 3 or 4. These solutions were transferred (100 µL) to 96-well microplates and, subsequently, 10 µL of the fermented juice was added under assay 6 conditions. After each assay, the plates were incubated for 3 h and then plated on MRS agar. The bacterial population was determined after 48 h of incubation.

### 2.6. Enteroaggregative Escherichia coli Infection in Larvae of Tenebrio molitor

The infection model using *T. molitor* larvae was used to determine the effectiveness of fermented or non-fermented juices in inhibiting infection provoked by enteroaggregative *Escherichia coli* (EAEC) 042 [27]. The juices were inoculated before (preventive) and after infection (treatment). In all experiments, the larvae (~100 mg) were anesthetized and disinfected (in ice and 70% alcohol, respectively) and randomly allocated to groups (n = 10/group). The infection was established by inoculation of 10 μL of standardized EAEC 042 suspension (OD_600nm_ = 0.1) at the membrane between the second and third abdominal annular segment (in the tail-head direction).

In the groups submitted to treatment, the 10-μL aliquots of the fermented and non-fermented juices (filtered through a 0.22 μm membrane) were inoculated 2 h after infection. On the other hand, to assess the preventive effect, aliquots of juices were administered 2 h before infection. Animals inoculated with phosphate-saline buffer (PBS, pH 7.4) were used for positive viability control. Larval survival was analyzed daily.

### 2.7. Statistical Analysis

The experiments were carried out in triplicate and in three independent tests. All results were expressed as mean values and were analyzed with consideration of the value of *p* < 0.05 as statistically significant. The data were analyzed using the GraphPad Prism^®^ (version 7.0) or Statistica software. Correlations were determined using Pearson’s coefficient (ρ) and classified as very strong (ρ ≥ 0.9), strong (0.7 ≤ ρ ≤ 0.89), moderate (0.5 ≤ ρ ≤ 0.69), weak (0.3 ≤ ρ ≤ 0.49) or negligible (ρ ≤ 0.29) [28]. Survival tests were analyzed using the Kaplan–Meier method and the log-rank test.

## 3. Results and Discussion

### 3.1. Analysis of Nutritional Composition of Bacuri Pulp

Bacuri is a typical fruit from the Legal Amazon region, and it is also found in other Brazilian biomes, such as the Cerrado biome. This fruit plays important economic roles in these areas; however it has been target of only few studies [14,19]. We performed the characterization of the pulp used in this study. The characterization of fruit pulps from Brazil, by determination of ash or water content is a matter of practical interest, since this property can interfere in the stability or useful life of the fruits produced. Even though they have a short harvest period, these fruits are often marketed in the form of pulp or dehydrated [29,30,31].

The results for moisture, ash content and macronutrients (proteins, lipids and carbohydrates) are shown in Table 2. Bacuri pulp showed high moisture content (81.67 ± 0.3%), a result similar to those observed for other fruits of the Brazilian cerrado (values ranging from 74.3 to 89.7%), such as cagaita (*Eugenia dysenterica*), mangaba (*Hancornia speciosa* Gomes) and marolo (*Annona crassiflora* Mart.) [30].

The ash content of bacuri pulp was 0.52 ± 0.02%, which corresponds to 2.83% of dry mass (DM). Regarding the macronutrients, the bacuri pulp had a higher amount of carbohydrates (13.81 ± 0.0% or 75.34% of DM), followed by proteins (3.9 ± 0.1% or 21.28% of DM) and lipids (0.10 ± 0.0 or 0.55% of DM). These results were also similar to those previously reported for bacuri pulp [32,33,34].

### 3.2. Growth and Viability after Storage of Lactobacilli strains in Platonia insignis Juice

**Figure 1:**
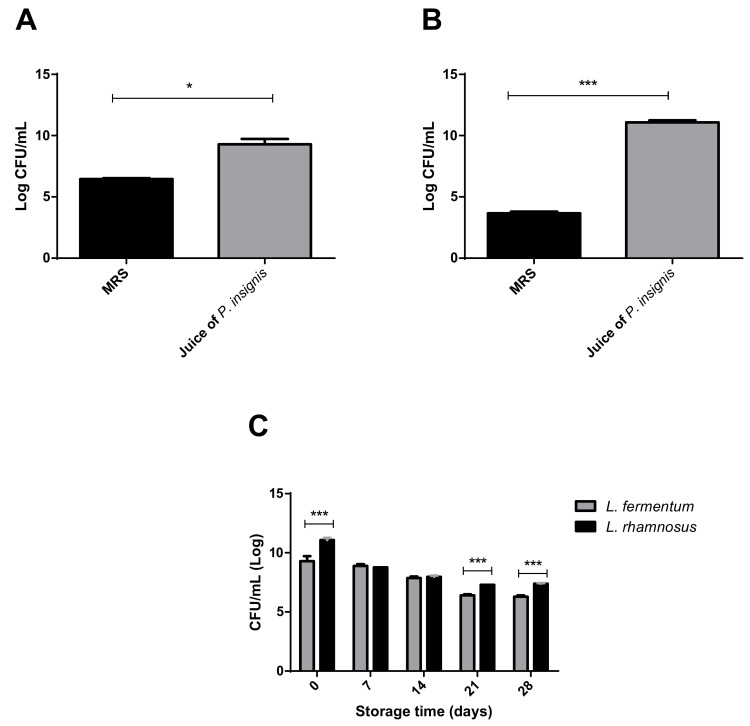
Evaluation of the growth of strains of *Lactobacillus* in the juice of *Platonia insignis* (bacuri). (**A**) Comparison of *Limosilactobacillus fermentum* ATCC 23,271 growth in MRS Agar and in *P. insignis* juice after 48 h of incubation. (**B**) Comparison of *Lacticaseibacillus rhamnosus* ATCC 9595 growth in MRS medium and in *P. insignis* juice after 48 h of incubation. (**C**) Comparison of the effect of storage on *P. insignis* juice on the population of *L. fermentum* ATCC 23,271 and *L. rhamnosus* ATCC 9595 in *Platonia insignis* juice. * Statistical differences with *p* < 0.05; *** Statistical differences with *p* < 0.001.

After evaluating the physical and chemical characteristics of the bacuri pulp, the next step of the work was to evaluate whether the lactobacilli strains had the capacity to grow in the juice of *P. insignis* (Figure 1). In both cases, a significantly higher growth was observed in bacuri juice (without the addition of nutritional supplements) than in MRS (*p* < 0.05). In the case of *L. fermentum* ATCC 23271, the average difference between growth in *P. insignis* juice and MRS was 2.84 Log CFU/mL (Figure 1A; *p* < 0.05); while the difference was 7.39 Log (Figure 1B; *p* < 0.001) for *L. rhamnosus* ATCC 9595.

Following this, the viability of each strain grown in the juice and stored at 4 °C for up to 28 days (Figure 1C) was analyzed. During this period, the lactobacilli remained viable with similar variation after 28 days (around 30% for both). However, the growth was significantly higher for *L. rhamnosus* ATCC 9595 (7.40 ± 0.04 Log CFU/mL) than *L. fermentum* ATCC 23,271 (6.29 ± 0.12 Log CFU/mL) (*p* < 0.0001). It is important to highlight that the viability levels at the end of the storage time were above the minimum limit (10⁶ CFU/mL; 15 to 30 days) for probiotic-containing products. Taken together, these data are evidence that the juice of *P. insignis* would be a good probiotic food matrix.

In general, these results are similar to those found for other juices [35,36]. The explanation for growth and high viability of these probiotic strains during storage in bacuri juice may be related to the chemical composition of this pulp with availability of amino acids (leucine, glutamine, arginine, alanine, valine and isoleucine), vitamins, minerals and carbohydrates (glucose, fructose and sucrose) that correspond with up to 50% of dry matter [33,37]. Further, the phenolic compounds from bacuri may be fermented by these bacteria and promote their growth [38].

### 3.3. Effects of Inoculum Density and Pulp Concentration on Bacterial Growth and Organic Acids Production

Based on the results of viability after storage, the strain *L. rhamnosus* ATCC 9595 was selected for further assays. *L. rhamnosus* ATCC 9595 was able to grow in all conditions evaluated by CCRD, with populations ranging from 7.52 ± 0.09 to 10.22 ± 0.09 Log CFU/mL). In these conditions the bacteria also produced organic acids, as seen by the decrease in initial pH to values ranging from 3.9 to 5.0 (Table 3). Using the results of bacterial growth and pH value, we calculated the G/pH ratios, to evaluate the most favorable conditions to obtain the higher levels of probiotic cells and organic acids [25,26]. The G/pH ratios ranged from 1.53 to 2.13 (Table 3).

The positive effects of inoculum concentration on G/pH ratio were also observed for the growth of *L. rhamnosus* ATCC 7469 in *Passiflora cincinnata* juice (Caatinga passion fruit). The study also reported similar values of bacterial growth (8–10 Log CFU/mL) compared to those observed in bacuri juice [26]. Regarding the pH variation, lactobacilli strains require slightly acidic pH for maximum growth [39]. The pH of bacuri pulp is around 3.12 to 3.48: these values corroborate with previous data reported by Silva et al. [40]. Thus, the pH of pulp was neutralized (pH~6.0) with 1 M NaOH, a food additive authorized by Brazilian legislation [24]. Neutralization of the pH allowed us to use the reduction of pH after the fermentation as an indication of organic acid production and growth [39,41,42].

**Figure 2:**
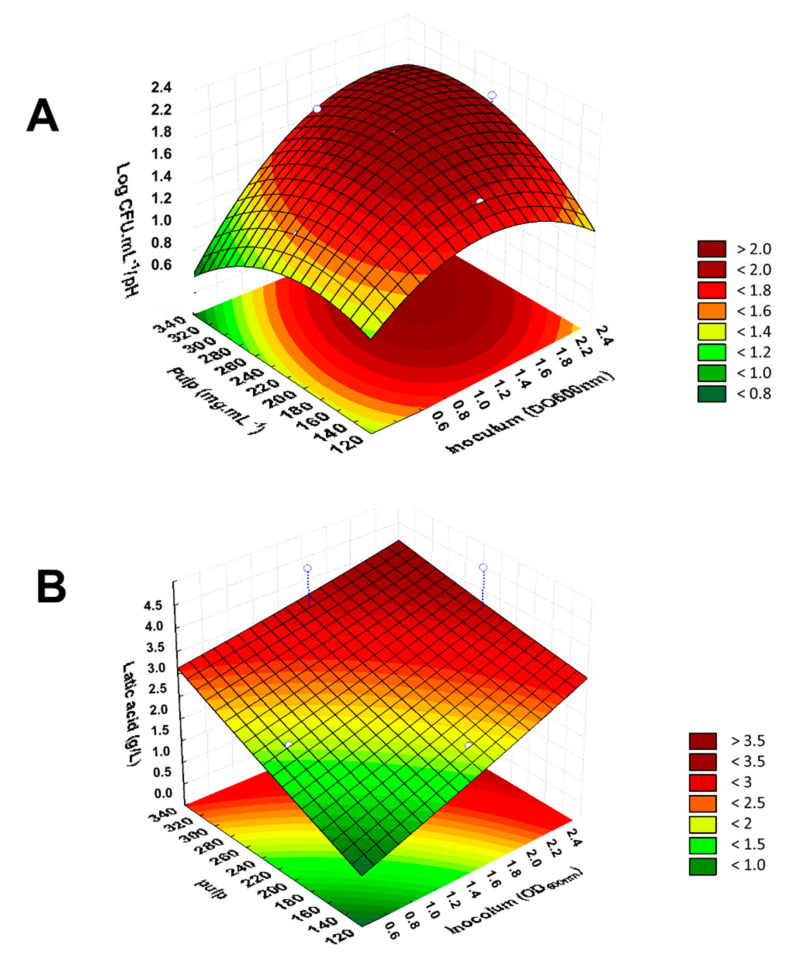
Effects of inoculum density and pulp concentration on the growth and production of organic acids by *L. rhamnosus* ATCC 9595 in *Platonia insignis* juice. (**A**) Response surface obtained for the Bacterial growth/pH ratio as a function of the studied variables. (**B**) Response surface obtained for the production of lactic acid as a function of the variables studied.

The G/pH ratio values ranged from 1.53 to 2.13 (Table 3). The surface response plot, shown in Figure 2A, illustrates the influence of the two selected independent variables (inoculum and pulp concentrations) in the values for G/pH ratio. A linearity coefficient (R^2^) of 0.87 was found and the curve can be described with the equation: z = 0.14767228670735 + 1.3205979104405x − 0.5332911196835x^2^ + 0.0081505369003327y − 0.000026303871055073y^2^ + 0.0027846725180107xy. The most favorable conditions were those used in assay 6 (G/pH = 2.13) and at the central points (assays 9 and 10; G/pH = 2.11 and 2.08). In these assays the same concentration of pulp (230.00 mg/mL) was used with different inoculum densities (2.33 and 1.55, respectively) (Table 3).

It was observed that the inoculum concentration is the main factor to obtain more favorable values of G/pH ratio (*p* < 0.05). This positive influence of the inoculum is evidenced by the analysis of Pearson’s coefficient (ρ), which indicates a strong correlation between the values of the inoculum and the G/pH ratios (ρ = 0.70); while the pulp concentration had a negligible correlation with these indexes (ρ = 0.10).

The positive effects of inoculum concentration on G/pH ratio were also observed for the optimization of *L. rhamnosus* ATCC 7469 in *Passiflora cincinnata* juice (Caatinga passion fruit). The study also reported similar values of bacterial growth (8–10 Log CFU/mL) compared to those observed in bacuri juice [26]. Regarding pH variation, the lactobacilli require slightly acidic pH for maximum growth [39].

### 3.4. Effects of Selected Conditions on Lactic Acid Production

The influence of the selected independent variables (inoculum and pulp concentrations) on the production of lactic acid by *L. rhamnosus* ATCC 9595 was also evaluated. The average initial concentration of lactic acid in *P. insignis* pulp was 0.18 ± 0.02 mg/g. The variation in the production of lactic acid was 1.32 to 4.14 g/L (Table 3), with the best yields being obtained in tests 6 and 8 (inoculum at 1.55 and pulp at 324.75 mg/mL) with concentrations of 4.14 and 3.98 g/L of lactic acid, respectively. The response surface generated with these data is represented in Figure 2B.

The independent variables on lactic acid production had no influence on lactic acid production (*p* > 0.05). Pearson’s coefficient analysis indicated that the inoculum concentration had a moderate correlation (ρ = 0.50) with acid production, while the concentration of the pulp has a weak correlation (ρ = 0.43). A moderate correlation was also observed between the production of lactic acid and bacterial growth (ρ = 0.66).

The production of organic acids by lactobacilli strains depends on the composition of the media [43,44]. In our study, the nitrogen and carbon sources available in bacuri juice were sufficient for synthesis of lactic acid by *L. rhamnosus* ATCC 9595. The levels of lactic acid by *L. rhamnosus* ATCC 9595 in bacuri juice were comparable to other *L. rhamnosus* strains grown in juices. For instance, *L. rhamnosus* HN001 produced a higher level of lactic acid (4.4 g/L) when cultivated in star fruit (*Averrhoa carambola*) juice [9].

We also evaluated the relation between bacterial growth and lactic acid production (G/(La) ratio) (Figure 2B). Again, the most favorable results were observed for the conditions of assays 6 and 8 (2.47 and 2.20) (Table 3). Although the variables did not significantly impact the G/(La) ratio, we could find a moderate correlation among G/pH and G/(La) ratios (ρ = 0.50). Based on our results, we decided to select the conditions used in assay 6 to perform the further evaluations.

### 3.5. The Cultivation of L. rhamnosus ATCC 9595 on Bacuri Juice Did Not Influence the Resistance towards Lysozyme and pH

Modulation of the gastrointestinal tract by probiotic bacteria depends on their ability to resist the adversities of the ecosystem (acidity and the presence of enzymes). Therefore, this characteristic should be evaluated for each probiotic candidate and their derived formulations [45]. *L. rhamnosus* ATCC 9595 is reported to completely tolerate pH values ≥ 2.5 in a simulated gastrointestinal fluid containing pepsin and NaCl [46].

**Figure 3:**
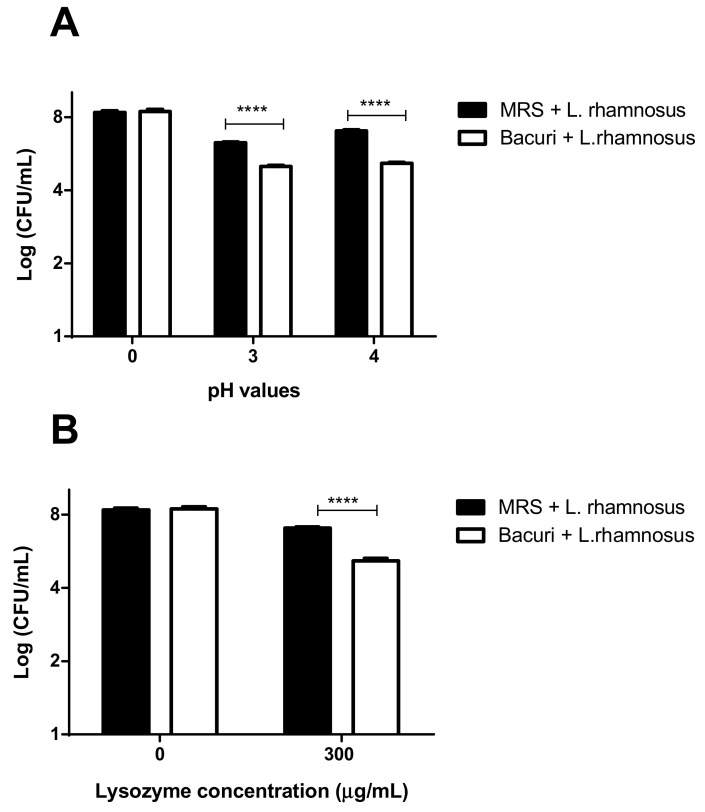
Test of tolerance to simulated conditions of the gastrointestinal tract. (**A**) pH tolerance test. (**B**) Lysozyme tolerance test. MRS + *L. rhamnosus* = *L. rhamnosus* ATCC 9595 grown on MRS; bacuri + *L. rhamnosus* = *L. rhamnosus* ATCC 9595 grown on bacuri juice. **** *p* < 0.0001.

Two assays were performed to assess whether the growth in *P. insignis* juice would alter the ability of *L. rhamnosus* ATCC 9595 to tolerate conditions found in the gastrointestinal tract (Figure 3). In both tests (pH tolerance and lysozyme), the bacteria showed similar results for growth in MRS and *P. insignis* juice (*p* > 0.05). CFU quantification ranged from 7.03 ± 0.05 to 5.15 ± 0.05 Log CFU/mL for pH 4, and from 6.27 ± 0.04 to 5.01 ± 0.05 Log CFU/mL for pH 3, when grown in MRS and *P. insignis* juice, respectively (Figure 3A). In the tolerance test, there was a bacterial growth of 7.05 ± 0.07 Log CFU/mL in MRS, whereas in bacuri juice the bacterial growth was 5.16 ± 0.13 Log CFU/mL (Figure 3B). In the condition tested (acidification or addition of lysozyme), *L. rhamnosus* ATCC 9595 showed similar viability profile when cultivated in MRS or bacuri juice.

### 3.6. Effects of Fermented and Non-Fermented Juices on Enteroaggregative Escherichia coli Infection in Larvae of Tenebrio molitor

**Figure 4:**
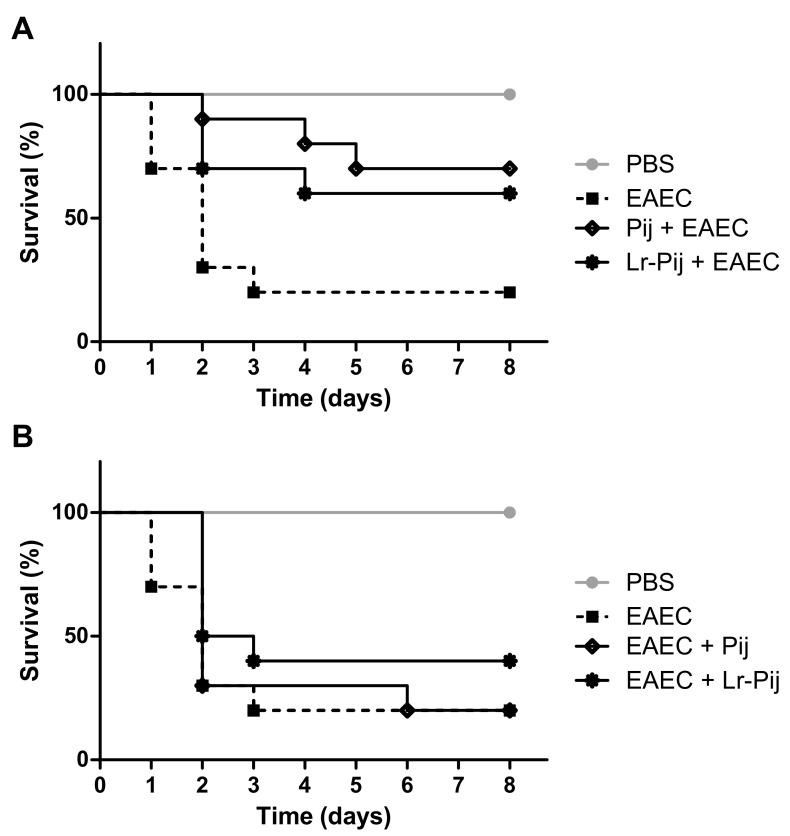
Effects of fermented and non-fermented juices on the infection induced by enteroaggregative *Escherichia coli* 042 in larvae of *Tenebrio molitor*. (**A**) Anti-infective effects of the pre-treatment with fermented and non-fermented juices on the infection induced by *E. coli*. (**B**) Anti-infective effects of the treatment with fermented and non-fermented juices on the infection induced by *E. coli*. PBS: phosphate-saline buffer; EAEC: enteroaggregative *Escherichia coli* 042; Pij: unfermented *Platonia insignis* juice; Lr-Pij: *Platonia insignis* juice fermented by *L. rhamnosus* ATCC9595.

Finally, the ability of fermented and non-fermented juice to alter the course of infection by EAEC 042 in *T. molitor* larvae was evaluated (Figure 4). This organism has been used as alternative model for study of microbial pathogenesis and prospection of anti-infective agents. In our assay, infected larvae without treatment had a median survival of 2 days, while uninfected larvae remained alive throughout the experimental period (8 days). The survival curves of these two groups were significantly different (*p* < 0.001).

The pre-treatment with supernatants of both fermented and non-fermented juices showed anti-infective effects with a significant increase in larval survival, in relation to untreated infected larvae (Figure 4A; *p* < 0.05). The survival percentages for animals pre-treated with juices at the end of the period were greater than 60%. The survival curves for infected and pre-treated larvae with the two types of juices did not show significant differences (*p* > 0.05). Further, we evaluated the effects of the inoculation of juices 2 h after infection by EAEC 042 (Figure 4B). Larvae treated with fermented juice showed a higher percentage of survival at the end of treatment (40%; median survival of 2.5 days) than the other infected groups (20% and median survival of 2 days).

Compounds with anti-microbial and anti-inflammatory potentials have been reported in bacuri pulp, which may explain these anti-infective effects observed for unfermented juice [47,48]. Similarly, some strains of *L. rhamnosus* exhibited inhibitory activity against *E. coli* infection [49,50]. For example, the administration of *L. rhamnosus* ATCC 9595 reduced the number of CFU for total Enterobacteriaceae in mice feces. This strain is also a promising antagonist agent towards *Candida albicans*, inhibiting the expression of virulence determinants (biofilm formation and filamentation) and protecting *Galleria mellonella* larvae [51,52].

## 4. Conclusions

Our data suggest that bacuri juice is an alternative substrate for cultivation of probiotic cultures, as it maintains the viability, growth and production of organic acids. The lactobacilli were stable after 28 days of refrigerated storage without the addition of any supplement. Importantly, the growth of *L. rhamnosus* ATCC 9595 in the juice did not alter its resistance in simulated conditions of the gastrointestinal tract. Further, the fermentation improved the anti-infective effects of juice towards infection of *T. molitor* larvae with a lethal dose of enteroaggregative *E. coli*. The results obtained in this research allowed the basis for the development of a non-dairy probiotic product from Bacuri juice.

## Figures and Tables

**Table 1:** Coded and decoded levels of independent variables.

Variables	−1.41	−1	0	+1	+1.41
Inoculum density (OD_600_)	0.77	1.00	1.55	2.10	2.33
Pulp concentration (mg/mL)	135.25	163.00	230.00	297.00	324.75

**Table 2:** Chemical composition of *P. insignis* pulp.

Attribute	Content (%)
Moisture	81.67 ± 0.3
Ash	0.52 ± 0.02
Proteins	3.9 ± 0.1
Lipids	0.10 ± 0.0
Carbohydrates	13.81 ± 0.0

**Table 3:** Effects of inoculum density and pulp concentration on growth and lactic acid production by *L. rhamnosus* ATCC 9595.

Assay	OD_600 nm_	Pulp (mg/mL)	Growth (CFU/mL)	Final pH	Lactic Acid (g/L)	G/pH Ratio	G/[La] Ratio
1	1.00	163.00	7.95 ± 0.10	4.80	1.32	1.66	6.02
2	1.00	297.00	7.52 ± 0.09	4.90	1.88	1.53	4.00
3	2.10	163.00	8.48 ± 0.04	4.90	1.94	1.73	4.37
4	2.10	297.00	9.49 ± 0.04	4.70	2.06	2.02	4.61
5	0.77	230.00	7.85 ± 0.02	5.00	1.97	1.57	3.98
6	2.33	230.00	10.22 ± 0.09	4.8	4.14	2.13	2.47
7	1.55	135.25	7.53 ± 0.06	3.90	2.12	1.93	3.55
8	1.55	324.75	8.78 ± 0.05	4.50	3.98	1.95	2.20
9	1.55	230.00	8.86 ± 0.04	4.20	2.48	2.11	3.57
10	1.55	230.00	8.72 ± 0.05	4.20	2.33	2.08	3.74

## Data Availability

The data presented in this study are available on request from the corresponding author.
